# Temperature Effect on Morphobiochemical Characters in Some Black Gram (*Vigna mungo*) Genotypes

**DOI:** 10.5402/2013/942868

**Published:** 2012-08-16

**Authors:** Manasi Dash, Dhara Shree

**Affiliations:** ^1^School of Biotech Sciences, Trident Academy of Creative Technology, Odisha Bhubaneswar 751024, India; ^2^Department of Biotechnology, AMIT, Odisha Bhubaneswar 751003, India

## Abstract

Lack of suitable varieties and genotypes of black gram with adaptation to local conditions is among the factors affecting its production. Efforts to genetically improve the crop mostly involve identifying important morphological descriptors followed by development of advanced breeding lines for locale-specific cultivars. The present day available black gram varieties have not been properly characterized for their thermo sensitiveness with respect to morphological and biochemical characters. Hence efforts were taken in the present research to study the effect of the temperature on these characters in seven black gram varieties over different development stages. We aimed at studying the effect of 3 temperature regimes for identifying suitable stress tolerant genotypes. High percent germination (87.2%), root length (3.68 cm), carbohydrate content (3.72 mg g^−1^ fresh tissue) among the genotypes was highest at 10°C–20°C temperature. High shoot length (13.39 cm), free amino acid content (3.73 mg g^−1^ fresh tissue), and protein content (9.54 mg g^−1^ fresh tissue) was found to be present when the genotypes were exposed to 20°C–30°C temperature. The black gram varieties J.L and PDU-1 performed best in all the temperature regimes over characters. Thus suitable varieties for all temperature regimes were identified using biochemical analysis.

## 1. Introduction

Black gram (*Vigna mungo*) is a tropical leguminous plant, which belongs to the asiatic *Vigna *species along with *V. radiata*, *V. trilobata*, *V. aconitifolia*, and *V. glaberecence.* It has high nutritive value and consists of high content of proteins, vitamins, and minerals.  *V. mungo *forms one of the important constituents in the dietary practices of the local communities. It is cultivated as fallow crop after rice cultivation in India. It is grown in various agroecological conditions and cropping systems with diverse agricultural practices. In recent years, there has been significant decline in its production in India. Lack of suitable varieties and genotypes with adaptation to local conditions is among the factors affecting its production. Efforts to genetically improve the crop are slow with only few efforts to identify important morphological descriptors and develop advanced breeding lines for locale-specific cultivars of this crop [1].

Stress is defined as an influence that is outside the normal range of homeostatic control in a given genotype [2]. Where a stress tolerance is exceeded, response mechanisms are activated, and where the stress is controlled, a new physiological state is established and homeostasis is reestablished. When the stress is removed, the plant may return to the original state or a new physiological state may be established [3]. Each plant species has its unique set of temperature requirements, which are optimum for proper growth and development. Low temperature is one of the abiotic stresses that cause crop failure. Many plants especially those which are native to warm habitat exhibit symptoms of injury when exposed to low nonfreezing temperatures. Low temperature stress induces considerable changes in biochemistry and physiology of plants. It generally causes a decrease in the entire metabolism and also in the photochemical steps of photosynthesis, which are interdependent on the biochemical phase and, as expected, more directly influenced by low temperatures [4, 5]. Studies of hardiness are essential in evaluating new cultivars of black gram. The general trend of research is to replace or to complete field testing by laboratory procedures. Identification of biochemical markers or some physiological indicators is advantageous for breeding and can be used as simple indicators of abiotic stress tolerance.

Here we aim at screening available genotypes of black gram by studying the effect of temperature regimes on various morphological and biochemical parameters at different time periods.

## 2. Materials and Methods

### 2.1. Plant Material and Growth Conditions

Healthy black gram seeds of 7 genotypes were collected from the Pulse Research Station, Berhampur, Odisha, India. These varieties are PU 30, SARALA, T9, OBG 17, B.3.3.8, PDU 1, and JAJPUR LOCAL. They were preserved in an incubator at 19°C. The seeds were selected for uniformity in size and surface sterilized with 0.1% w/v mercuric chloride (HgCl_2_) for 5 min followed by thorough washing with sterile double-distilled water for 4-5 times. The sterilized seeds were soaked overnight. These soaked seeds were kept in sterilized petriplates containing soaked filter paper in such a way that each petriplate contained 15 seeds.

The effect of different temperature duration on various morphological and biochemical characters was studied. For the present research three temperature regimes **A** (0°C to 10°C), **B** (10°C to 20°C), and **C **(20°C to 30°C) were chosen. To maintain 0°C to 10°C temperature the petriplates containing the seeds were kept in an ice basket for germination. For 10°C to 20°C, the seeds were allowed to germinate in the culture room where the average room temperature was maintained. To study the effect of 20°C to 30°C temperature, the seeds were germinated at room temperature.

### 2.2. Growth Study

To study the effect of temperature on morphological characters a number of parameters were studied on 3rd day and 10th day of sowing the seeds. The different parameters studied were percent germination, shoot length, root length, fresh weight, and dry weight of seedlings. The percent germination was calculated by counting the number of seeds that have sprouted in each replication. The fresh plant weight was recorded by taking average of 5 plants from each replication. These were then kept in hot air oven at 60°C for 15 min, and their dry weight was recorded.

### 2.3. Biochemical Analysis

For sugar estimation 500 mg of plant sample was weighted and ground with 50 mL of 80% ethanol (v/v). The homogenate was centrifuged twice each time with 5 mL of 80% ethanol. Supernatant was collected in a beaker and evaporated in water bath. After evaporation of all the supernatant, 10 mL of double-distilled water was added to dissolve the sugar left in the beaker. The dissolved sugar was kept in 5 mL bottle at 20°C. The DNS (dinitrosalicylic acid reagent) method described by Sadasivam and Manickam [6] was followed for reducing sugar estimation.

For extraction of protein, 1 gm of sample was ground in 5 mL phosphate buffer (50 mM, pH 7.5). The homogenate was centrifuged at 8000 rpm for 20 min for five times. All supernatant was collected and made up to 50 mL volume with phosphate buffer. 1 mL of this was mixed with 1 mL of TCA (20%) and incubated for 30 min at 4°C. It was then centrifuged at 8000 rpm for 20 min. The supernatant was discarded, and the pellet was washed with cold acetone and again centrifuged at 8000 rpm for 20 min at 4°C. The resultant pellet was dissolved in 5 mL NaOH (0.1N) and supernatant was discarded. This was used for determining the protein content.

The total soluble protein of the seedlings was estimated as described by Lowry et al. [7] using BSA as standard. To estimate the amino acid content 0.5 gm of plant sample was ground with small quantity of acid-washed sand. To this homogenate 5–10 mL of 80% ethanol (v/v) was added and centrifuged. The supernatant was collected, and 80% ethanol (v/v) was added and centrifuged. The procedure was repeated five times, and supernatant was collected and pooled together. Total volume was made up to 50 mL with 80% ethanol. 5–10 mL of the supernatant was taken and evaporated in water bath. The residue was finally dissolved in 5 mL citrate buffer (0.2 M, pH 5.0). Amino acid content was estimated by the method of Moore and Stein [8] using L-glycine as standard.

### 2.4. Data Analysis

All the experiments were repeated three times. Each replication consisted of average of 15 seeds per genotype per character per temperature regime, and observations were recorded. Analysis of variance (ANOVA) for each character among the genotypes was carried out as per the Student's *t*-test.

## 3. Results

### 3.1. Effect of 0°C –10°C Temperature

**Figure 1:**
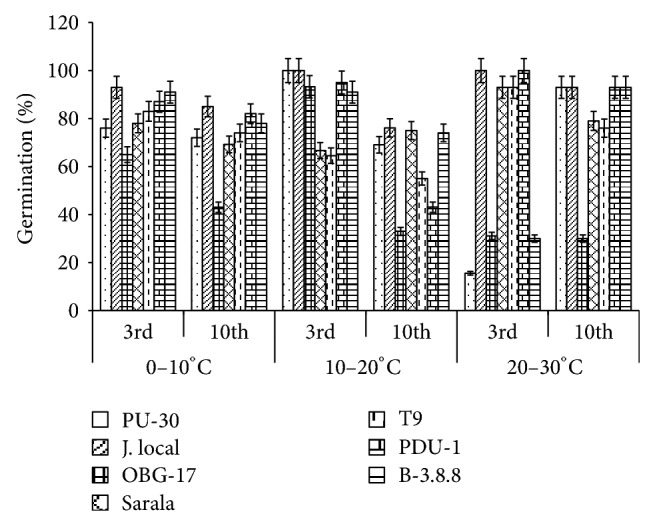
Effect of temperature on germination % of black gram. All the experiments were repeated three times. Each replication consisted of average of 15 seeds per genotype per character per temperature regime, and observations were recorded. Data were recorded on 3rd day and 10th day of germination of seedlings. Analysis of variance (ANOVA) for each character among the genotypes was carried out as per the Student's *t*-test.

**Figure 2:**
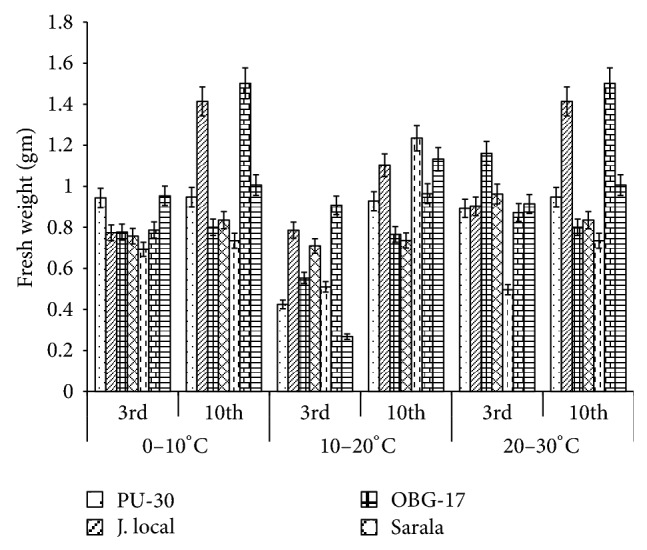
Effect of temperature on fresh weight of black gram. All the experiments were repeated three times. Each replication consisted of average of 15 seeds per genotype per character per temperature regime, and observations were recorded. Data were recorded on 3rd day and 10th day of germination of seedlings. Analysis of variance (ANOVA) for each character among the genotypes was carried out as per the Student's *t*-test.

The genotypes recorded high germination % (Figure 1) on 3rd day (81.85%). Highest shoot length was recorded by T-9 (2.94 cm) and J-L (14.25 cm) on 3rd and 10th day of germination, respectively. J.L recorded the highest root length (Figure 5) on 3rd day (2.2 cm) as well as on 10th day (3.84 cm). The genotypes recorded high mean root length on 10th day (1.75 cm). P.D.U-1 showed the highest fresh weight (1.5 gm) on 10th day while PU-30 recorded the highest fresh weight of 0.94 gm on 3rd day (Figure 2). Genotypes recorded high mean dry weight on 10th day (0.06 gm). O.B.G-17 and J.L recorded the highest amino acid content of 0.95 mg g^−1^ fresh weight (Figure 7) on 3rd day. The genotypes recorded high mean carbohydrate content (3.72 mg g^−1^ fresh weight), amino acid content (0.87 mg g^−1^ fresh weight), and protein content (9.28 mg g^−1^ fresh weight) on 3rd day.

### 3.2. Effect of 10°C–20°C Temperature

**Figure 3:**
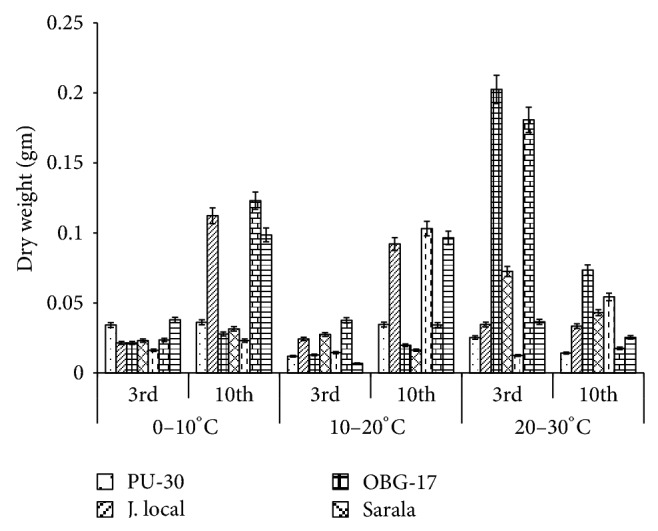
Effect of temperature on dry weight of black gram genotypes. All the experiments were repeated three times. Each replication consisted of average of 15 seeds per genotype per character per temperature regime, and observations were recorded. Data were recorded on 3rd day and 10th day of germination of seedlings. Analysis of variance (ANOVA) for each character among the genotypes was carried out as per the Student's *t*-test.

The genotypes recorded high germination % on 3rd day (87.2%) and only 67.74% on 10th day. The mean shoot length (11.48 cm) and root length of the genotypes were higher (3.68 cm) on 10th day. The black gram genotype P.D.U.1 showed the highest fresh weight of 0.90 gm on 3rd day and T-9 the highest (1.23 gm) on 10th day (Figure 2). The genotypes exhibited higher mean fresh weight (0.98 gm) on 10th day. The mean dry weight of the varieties of black gram (Figure 3) was high on 10th day of germination (0.05 gm). O.B.G-17 recorded the highest carbohydrate content (4 mg g^−1^ fresh tissue) on 3rd day while on 10th day the black gram variety T9 recorded the highest amount of carbohydrate, that is, 3 mg g^−1^ fresh tissue (Figure 6). The mean amino acid content of the black gram genotypes (Figure 7) recorded highest on 3rd day (0.51 mg g^−1^ fresh tissue). The mean protein content (Figure 8) of the black gram genotypes recorded highest on 3rd day (6.28 mg g^−1^ fresh tissue).

### 3.3. Effect of 20°C–30°C Temperature

**Figure 4:**
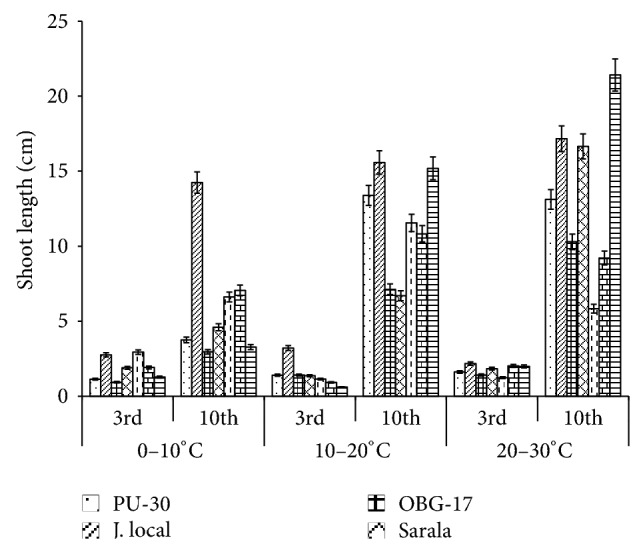
Effect of temperature on shoot length of black gram genotypes. All the experiments were repeated three times. Each replication consisted of average of 15 seeds per genotype per character per temperature regime, and observations were recorded. Data were recorded on 3rd day and 10th day of germination of seedlings. Analysis of variance (ANOVA) for each character among the genotypes was carried out as per the Student's *t*-test.

**Figure 5:**
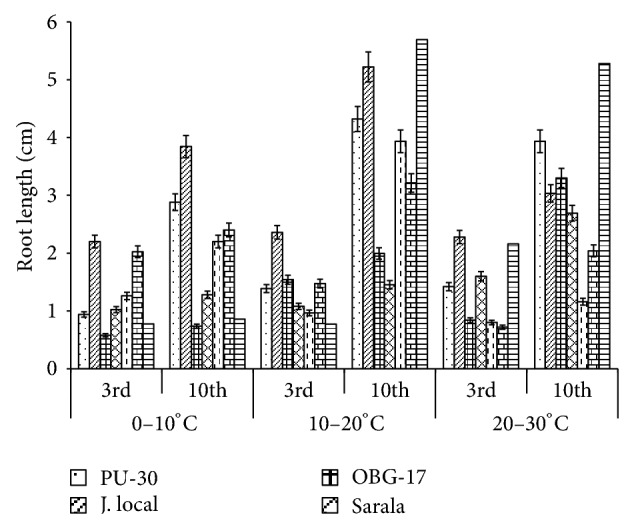
Effect of temperature on root length of black gram genotypes. All the experiments were repeated three times. Each replication consisted of average of 15 seeds per genotype per character per temperature regime, and observations were recorded. Data were recorded on 3rd day and 10th day of germination of seedlings. Analysis of variance (ANOVA) for each character among the genotypes was carried out as per the Student's *t*-test.

**Figure 6:**
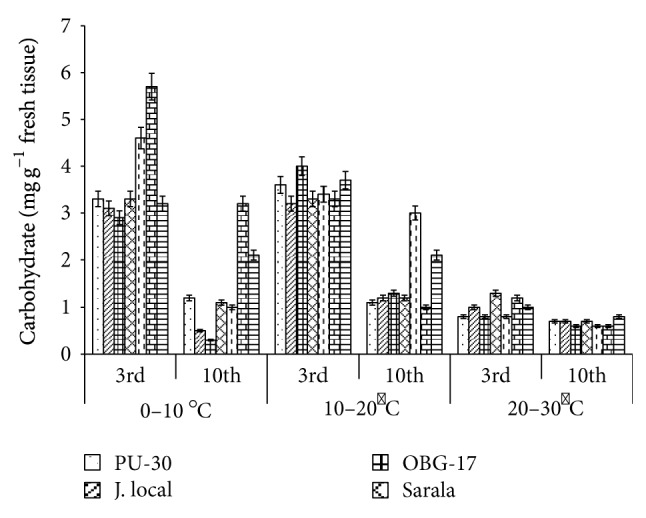
Effect of temperature on carbohydrate content of black gram genotypes. All the experiments were repeated three times. Each replication consisted of average of 15 seeds per genotype per character per temperature regime, and observations were recorded. Data were recorded on 3rd day and 10th day of germination of seedlings. Analysis of variance (ANOVA) for each character among the genotypes was carried out as per the Student's *t*-test.

**Figure 7:**
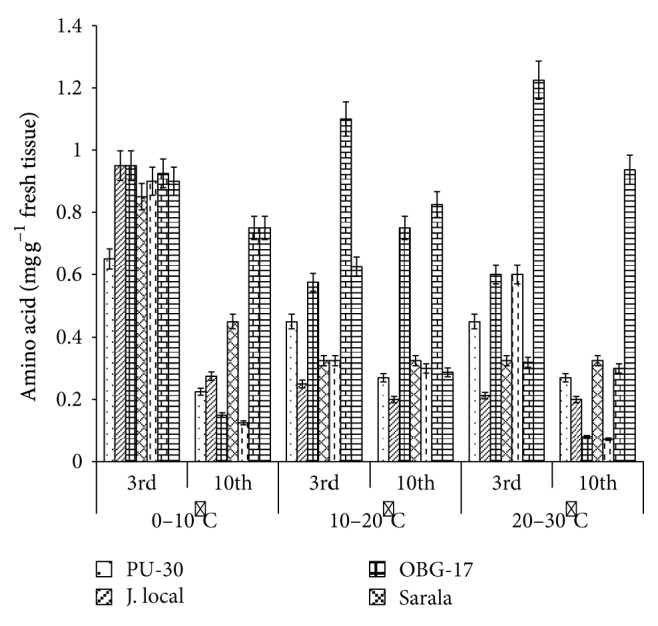
Effect of temperature on amino acid content of black gram genotypes. All the experiments were repeated three times. Each replication consisted of average of 15 seeds per genotype per character per temperature regime, and observations were recorded. Data were recorded on 3rd day and 10th day of germination of seedlings. Analysis of variance (ANOVA) for each character among the genotypes was carried out as per the Student's *t*-test.

**Figure 8:**
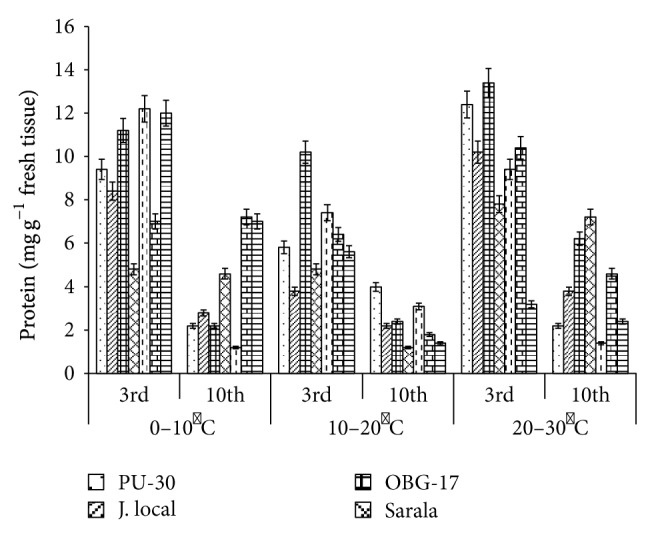
Effect of temperature on protein content of black gram genotypes. All the experiments were repeated three times. Each replication consisted of average of 15 seeds per genotype per character per temperature regime, and observations were recorded. Data were recorded on 3rd day and 10th day of germination of seedlings. Analysis of variance (ANOVA) for each character among the genotypes was carried out as per the Student's *t*-test.

The mean germination was only 66.09% on 3rd day which increased to 79.54% on 10th day of sowing the black gram seeds at temperature 20°C–30°C (Figure 1). The mean shoot length of the genotypes (Figure 4) was higher (13.39 cm) on 10th day than on 3rd day. The mean root length of the genotypes was higher (3.06 cm) on 10th day than on 3rd day (1.40 cm). On 3rd day black gram variety Sarala showed the highest carbohydrate content of 1.3 mg g^−1^ fresh tissue. The mean carbohydrate content of the black gram genotypes recorded highest on 3rd day (0.98 mg g^−1^ fresh tissue) which decreased on 10th day to 0.67 mg g^−1^ fresh tissue (Figure 6). The mean amino acid content of the black gram genotypes recorded highest on 3rd day (0.53 mg g^−1^ fresh tissue). The mean protein content of the black gram genotypes (Figure 8) recorded highest on 3rd day (9.54 mg g^−1^ fresh tissue). On 3rd day PU-30 showed the highest protein content with 12.4 mg g^−1^ fresh tissue and Sarala highest with 7.2 mg g^−1^ fresh tissue on 10th day.

## 4. Discussion

Black gram is a thermosensitive crop. The temperature during germination, seedling, and reproductive stage is very critical. A number of varieties are available which have high yield potential. But the potential is not realized to the maximum due to its thermosensitiveness. The present day available varieties have not been properly characterized for their thermosensitiveness with respect to morphological and biochemical characters. The differential response of genotypes to temperature stress is considered as the basis for the observed genetic variability. The data revealed significant difference among the genotypes for various morphological and biochemical characters. Any morphological or biochemical adaptation of a genotype is a consequence of gene expression, and the gene product brings about the required metabolic changes for adaptation. In the present study the exposure of the seven varieties to three different temperature regimes, that is, 0°C−10°C, 10°C−20°C, and 20°C−30°C, resulted in variable morphobiochemical characters. This variation may be due to variation in stress adaptive mechanism among the genotypes.

It has been observed that the prolonged exposure of leaves to low temperature resulted in selective inactivation of the oxygen evolving-system in cucumber [9–13], bean [14], and tomato [15]. According to Weis [16], the acclimation of the photosynthetic apparatus to high temperature could be perceived as a long-term response of the leaves to changes in the temperature regime for several days or weeks. However, significant changes for some hours, in diurnal temperature, can often be observed as well. Acclimation involves both short-term chemical, molecular, and physiological responses and long-term physiological, structural, and morphological modifications [17]. In most cases the adaptive mechanisms to different thermal regimes could be considered as compensatory, because they enable plants to buffer the effect of the temperature shift on their metabolic systems [18]. As a result, the amount of specific components compensating the temperature effect on the rate of a given reaction changes. The protein synthesizing system plays a crucial role in plant acclimation processes. It has been hypothesized that particular proteins whose synthesis is induced by stress conditions are critical for the survival in that stress [19, 20]. Pretreatments which lead to acquisition of thermotolerance are conditions under which heat shock proteins (HSPs) are synthesized [21–23]. During HS, these HSPs could interact with other proteins to prevent their aggregation and facilitate reassembly of functional structure [24]. This may explain the occurrence of very high protein content under low temperature conditions, that is, 0°C−10°C. The present research indicates sufficient genetic variability existing within the black gram genotypes from tolerance to temperature stress. The black gram varieties J.L and PDU-1 performed best at all the temperature durations over characters.
